# Association of BMI and Cognitive Performance in the Diabetes Prevention Program Outcomes Study

**DOI:** 10.1002/oby.70031

**Published:** 2025-09-28

**Authors:** José A. Luchsinger, Qing Pan, William C. Knowler, Medha Munshi, Karol Watson, Kishore M. Gadde, Mathias Schlögl, Owen T. Carmichael, DPP Research Group, George A. Bray, Kishore M. Gadde, Iris W. Culbert, Jennifer Arceneaux, Annie Chatellier, Amber Dragg, Catherine M. Champagne, Crystal Duncan, Barbara Eberhardt, Frank Greenway, Fonda G. Guillory, April A. Herbert, Michael L. Jeffirs, Betty M. Kennedy, Erma Levy, Monica Lockett, Jennifer C. Lovejoy, Laura H. Morris, Lee E. Melancon, Donna H. Ryan, Deborah A. Sanford, Kenneth G. Smith, Lisa L. Smith, Richard T. Tulley, Paula C. Vicknair, Donald Williamson, Jeffery J. Zachwieja, Kenneth S. Polonsky, Janet Tobian, David A. Ehrmann, Margaret J. Matulik, Karla A. Temple, Bart Clark, Kirsten Czech, Catherine DeSandre, Brittnie Dotson, Ruthanne Hilbrich, Wylie McNabb, Ann R. Semenske, Celeste C. Thomas, Jose F. Caro, Kevin Furlong, Barry J. Goldstein, Pamela G. Watson, Kellie A. Smith, Jewel Mendoza, Marsha Simmons, Wendi Wildman, Renee Liberoni, John Spandorfer, Constance Pepe, Richard P. Donahue, Ronald B. Goldberg, Ronald Prineas, Jeanette Calles, Anna Giannella, Patricia Rowe, Juliet Sanguily, Paul Cassanova‐Romero, Sumaya Castillo‐Florez, Hermes J. Florez, Rajesh Garg, Lascelles Kirby, Olga Lara, Carmen Larreal, Valerie McLymont, Jadell Mendez, Arlette Perry, Patrice Saab, Bertha Veciana, Steven M. Haffner, Helen P. Hazuda, Maria G. Montez, Kathy Hattaway, Juan Isaac, Carlos Lorenzo, Arlene Martinez, Monica Salazar, Tatiana Walker, Dana Dabelea, Richard F. Hamman, Patricia V. Nash, Sheila C. Steinke, Lisa Testaverde, Jennifer Truong, Denise R. Anderson, Larry B. Ballonoff, Alexis Bouffard, Brian Bucca, B. Ned Calonge, Lynne Delve, Martha Farago, James O. Hill, Shelley R. Hoyer, Tonya Jenkins, Bonnie T. Jortberg, Dione Lenz, Marsha Miller, Thomas Nilan, Leigh Perreault, David W. Price, Judith G. Regensteiner, Emily B. Schroeder, Helen Seagle, Carissa M. Smith, Brent VanDorsten, Edward S. Horton, Medha Munshi, Kathleen E. Lawton, Catherine S. Poirier, Kati Swift, Ronald A. Arky, Marybeth Bryant, Jacqueline P. Burke, Enrique Caballero, Karen M. Callaphan, Barbara Fargnoli, Therese Franklin, Om P. Ganda, Ashley Guidi, Mathew Guido, Alan M. Jacobsen, Lyn M. Kula, Margaret Kocal, Lori Lambert, Kathleen E. Lawton, Sarah Ledbury, Maureen A. Malloy, Roeland J.W. Middelbeek, Maryanne Nicosia, Cathryn F. Oldmixon, Jocelyn Pan, Marizel Quitingon, Riley Rainville, Stacy Rubtchinsky, Ellen W. Seely, Jessica Sansoucy, Dana Schweizer, Donald Simonson, Fannie Smith, Caren G. Solomon, Jeanne Spellman, James Warram, Steven E. Kahn, Brenda K. Montgomery, Basma Fattaleh, Celeste Colegrove, Wilfred Fujimoto, Robert H. Knopp, Edward W. Lipkin, Michelle Marr, Ivy Morgan‐Taggart, Anne Murillo, Kayla O'Neal, Dace Trence, Lonnese Taylor, April Thomas, Elaine C. Tsai, Samuel Dagogo‐Jack, Abbas E. Kitabchi, Mary E. Murphy, Laura Taylor, Jennifer Dolgoff, William B. Applegate, Michael Bryer‐Ash, Debra Clark, Sandra L. Frieson, Uzoma Ibebuogu, Raed Imseis, Helen Lambeth, Lynne C. Lichtermann, Hooman Oktaei, Harriet Ricks, Lily M.K. Rutledge, Amy R. Sherman, Clara M. Smith, Judith E. Soberman, Beverly Williams‐Cleaves, Avnisha Patel, Ebenezer A. Nyenwe, Ethel Faye Hampton, Boyd E. Metzger, Mark E. Molitch, Amisha Wallia, Mariana K. Johnson, Daphne T. Adelman, Catherine Behrends, Michelle Cook, Marian Fitzgibbon, Mimi M. Giles, Deloris Heard, Cheryl K.H. Johnson, Diane Larsen, Anne Lowe, Megan Lyman, David McPherson, Samsam C. Penn, Thomas Pitts, Renee Reinhart, Susan Roston, Pamela A. Schinleber, Matthew O'Brien, Monica Hartmuller, David M. Nathan, Charles McKitrick, Heather Turgeon, Mary Larkin, Marielle Mugford, Kathy Abbott, Ellen Anderson, Laurie Bissett, Kristy Bondi, Enrico Cagliero, Jose C. Florez, Linda Delahanty, Valerie Goldman, Elaine Grassa, Lindsery Gurry, Kali D'Anna, Fernelle Leandre, Peter Lou, Alexandra Poulos, Elyse Raymond, Valerie Ripley, Christine Stevens, Beverly Tseng, Kathy Chu, Nopporn Thangthaeng, Jerrold M. Olefsky, Elizabeth Barrett‐Connor, Sunder Mudaliar, Maria Rosario Araneta, Mary Lou Carrion‐Petersen, Karen Vejvoda, Sarah Bassiouni, Madeline Beltran, Lauren N. Claravall, Jonalle M. Dowden, Steven V. Edelman, Pranav Garimella, Robert R. Henry, Javiva Horne, Marycie Lamkin, Simona Szerdi Janesch, Diana Leos, William Polonsky, Rosa Ruiz, Jean Smith, Jennifer Torio‐Hurley, F. Xavier Pi‐Sunyer, Blandine Laferrere, Jane E. Lee, Susan Hagamen, David B. Allison, Nnenna Agharanya, Nancy J. Aronoff, Maria Baldo, Jill P. Crandall, Sandra T. Foo, Kim Kelly‐Dinham, Jose A. Luchsinger, Carmen Pal, Kathy Parkes, Mary Beth Pena, Ellen S. Rooney, Gretchen E.H. Van Wye, Kristine A. Viscovich, Mary de Groot, David G. Marrero, Kieren J. Mather, Melvin J. Prince, Susie M. Kelly, Marcia A. Jackson, Gina McAtee, Paula Putenney, Ronald T. Ackermann, Carolyn M. Cantrell, Yolanda F. Dotson, Edwin S. Fineberg, Megan Fultz, John C. Guare, Angela Hadden, James M. Ignaut, Marion S. Kirkman, Erin O'Kelly Phillips, Kisha L Pinner, Beverly D. Porter, Paris J. Roach, Nancy D. Rowland, Madelyn L. Wheeler, Vanita Aroda, Michelle Magee, Robert E. Ratner, Michelle Magee, Gretchen Youssef, Sue Shapiro, Natalie Andon, Catherine Bavido‐Arrage, Geraldine Boggs, Marjorie Bronsord, Ernestine Brown, Holly Love Burkott, Wayman W. Cheatham, Susan Cola, Cindy Evans, Peggy Gibbs, Tracy Kellum, Lilia Leon, Milvia Lagarda, Claresa Levatan, Milajurine Lindsay, Asha K. Nair, Jean Park, Maureen Passaro, Angela Silverman, Gabriel Uwaifo, Debra Wells‐Thayer, Renee Wiggins, Mohammed F. Saad, Karol Watson, Christine Darwin, Preethi Srikanthan, Tamara Horwich, Adrian Casillas, Arleen Brown, Maria Budget, Sujata Jinagouda, Medhat Botrous, Anthony Sosa, Sameh Tadros, Khan Akbar, Claudia Conzues, Perpetua Magpuri, Carmen Muro, Noemi Neira, Kathy Ngo, Michelle Chan, Veronica Villarreal, Amer Rassam, Debra Waters, Kathy Xapthalamous, Julio V. Santiago, Samuel Dagogo‐Jack, Neil H. White, Angela L. Brown, Samia Das, Prajakta Khare‐Ranade, Tamara Stich, Ana Santiago, Edwin Fisher, Emma Hurt, Tracy Jones, Michelle Kerr, Lucy Ryder, Cormarie Wernimont, Sherita Hill Golden, Christopher D. Saudek, Vanessa Bradley, Emily Sullivan, Tracy Whittington, Caroline Abbas, Adrienne Allen, Frederick L. Brancati, Sharon Cappelli, Jeanne M. Clark, Jeanne B. Charleston, Janice Freel, Katherine Horak, Alicia Greene, Dawn Jiggetts, Deloris Johnson, Hope Joseph, Kimberly Loman, Nestoras Mathioudakis, Henry Mosley, John Reusing, Richard R. Rubin, Alafia Samuels, Thomas Shields, Shawne Stephens, Kerry J. Stewart, LeeLana Thomas, Evonne Utsey, Paula Williamson, David S. Schade, Karwyn S. Adams, Janene L. Canady, Carolyn Johannes, Claire Hemphill, Penny Hyde, Leslie F. Atler, Patrick J. Boyle, Mark R. Burge, Lisa Chai, Kathleen Colleran, Ateka Fondino, Ysela Gonzales, Doris A. Hernandez‐McGinnis, Patricia Katz, Carolyn King, Julia Middendorf, Amer Rassam, Sofya Rubinchik, Willette Senter, Debra Waters, Jill Crandall, Harry Shamoon, Janet O. Brown, Gilda Trandafirescu, Danielle Powell, Norica Tomuta, Elsie Adorno, Liane Cox, Helena Duffy, Samuel Engel, Allison Friedler, Angela Goldstein, Crystal J. Howard‐Century, Jennifer Lukin, Stacey Kloiber, Nadege Longchamp, Helen Martinez, Dorothy Pompi, Jonathan Scheindlin, Elissa Violino, Elizabeth A. Walker, Judith Wylie‐Rosett, Elise Zimmerman, Joel Zonszein, Trevor Orchard, Elizabeth Venditti, Rena R. Wing, Susan Jeffries, Gaye Koenning, M. Kaye Kramer, Marie Smith, Susan Barr, Catherine Benchoff, Miriam Boraz, Lisa Clifford, Rebecca Culyba, Marlene Frazier, Ryan Gilligan, Stephanie Guimond, Susan Harrier, Louann Harris, Andrea Kriska, Qurashia Manjoo, Monica Mullen, Alicia Noel, Amy Otto, Jessica Pettigrew, Bonny Rockette‐Wagner, Debra Rubinstein, Linda Semler, Cheryl F. Smith, Valarie Weinzierl, Katherine V. Williams, Tara Wilson, Bonnie Gillis, Marjorie K. Mau, Narleen K. Baker‐Ladao, John S. Melish, Richard F. Arakaki, Renee W. Latimer, Mae K. Isonaga, Ralph Beddow, Nina E. Bermudez, Lorna Dias, Jillian Inouye, Kathy Mikami, Pharis Mohideen, Sharon K. Odom, Raynette U. Perry, Robin E. Yamamoto, William C. Knowler, Robert L. Hanson, Harelda Anderson, Norman Cooeyate, Charlotte Dodge, Mary A. Hoskin, Carol A. Percy, Alvera Enote, Camille Natewa, Kelly J. Acton, Vickie L. Andre, Rosalyn Barber, Shandiin Begay, Peter H. Bennett, Mary Beth Benson, Evelyn C. Bird, Brenda A. Broussard, Brian C. Bucca, Marcella Chavez, Sherron Cook, Jeff Curtis, Tara Dacawyma, Matthew S. Doughty, Roberta Duncan, Cyndy Edgerton, Jacqueline M. Ghahate, Justin Glass, Martia Glass, Dorothy Gohdes, Wendy Grant, Ellie Horse, Louise E. Ingraham, Merry Jackson, Priscilla Jay, Roylen S. Kaskalla, Karen Kavena, David Kessler, Kathleen M. Kobus, Jonathan Krakoff, Jason Kurland, Catherine Manus, Cherie McCabe, Sara Michaels, Tina Morgan, Yolanda Nashboo, Julie A. Nelson, Steven Poirier, Evette Polczynski, Christopher Piromalli, Mike Reidy, Jeanine Roumain, Debra Rowse, Robert J. Roy, Sandra Sangster, Janet Sewenemewa, Miranda Smart, Chelsea Spencer, Darryl Tonemah, Rachel Williams, Charlton Wilson, Michelle Yazzie, Raymond Bain, Sarah Fowler, Marinella Temprosa, Michael D. Larsen, Kathleen Jablonski, Tina Brenneman, Sharon L. Edelstein, Solome Abebe, Julie Bamdad, Melanie Barkalow, Joel Bethepu, Tsedenia Bezabeh, Anna Bowers, Nicole Butler, Jackie Callaghan, Caitlin E. Carter, Costas Christophi, Gregory M. Dwyer, Mary Foulkes, Yuping Gao, Robert Gooding, Adrienne Gottlieb, Kristina L. Grimes, Nisha Grover‐Fairchild, Lori Haffner, Heather Hoffman, Steve Jones, Tara L. Jones, Richard Katz, Preethy Kolinjivadi, John M. Lachin, Yong Ma, Pamela Mucik, Robert Orlosky, Qing Pan, Susan Reamer, James Rochon, Alla Sapozhnikova, Hanna Sherif, Charlotte Stimpson, Ashley Hogan Tjaden, Fredricka Walker‐Murray, Audrey McMaster, Rhea Mundra, Hannah Rapoport, Nolan Kuenster, Lifestyle Resource Core, Elizabeth M. Venditti, Andrea M. Kriska, Linda Semler, Valerie Weinzierl, Santica Marcovina, F. Alan Aldrich, Jessica Harting, John Albers, Greg Strylewicz, Robert Janicek, Anthony Killeen, Deanna Gabrielson, R. Eastman, Judith Fradkin, Sanford Garfield, Christine Lee, Edward Gregg, Ping Zhang, Carotid Ultrasound, Dan O'Leary, Gregory Evans, Coronary Artery Calcification Reading Center, Matthew Budoff, Chris Dailing, CT Scan Reading Center, Elizabeth Stamm, Ann Schwartz, Caroline Navy, Lisa Palermo, Pentti Rautaharju, Ronald J. Prineas, Teresa Alexander, Charles Campbell, Sharon Hall, Yabing Li, Margaret Mills, Nancy Pemberton, Farida Rautaharju, Zhuming Zhang, Elsayed Z. Soliman, Julie Hu, Susan Hensley, Lisa Keasler, Tonya Taylor, Barbara Blodi, Ronald Danis, Matthew Davis, Larry Hubbard, Ryan Endres, Deborah Elsas, Samantha Johnson, Dawn Myers, Nancy Barrett, Heather Baumhauer, Wendy Benz, Holly Cohn, Ellie Corkery, Kristi Dohm, Amitha Domalpally, Vonnie Gama, Anne Goulding, Andy Ewen, Cynthia Hurtenbach, Daniel Lawrence, Kyle McDaniel, Jeong Pak, James Reimers, Ruth Shaw, Maria Swift, Pamela Vargo, Sheila Watson, Neurocognitive Assessment Group, Jose A. Luchsinger, Jennifer Manly, Elizabeth Mayer‐Davis, Robert R. Moran, Ted Ganiats, Kristin David, Andrew J. Sarkin, Erik Groessl, Naomi Katzir, Helen Chong, William H. Herman, Michael Brändle, Morton B. Brown, Jose C. Florez, David Altshuler, Liana K. Billings, Ling Chen, Maegan Harden, Robert L. Hanson, William C. Knowler, Toni I. Pollin, Alan R. Shuldiner, Kathleen Jablonski, Paul W. Franks, Marie‐France Hivert

**Affiliations:** ^1^ Department of Medicine and Epidemiology Columbia University Irving Medical Center New York New York USA; ^2^ Department of Biostatistics and Bioinformatics, Milken School of Public Health George Washington University Rockville Maryland USA; ^3^ Department of Medicine Joslin Diabetes Center, Beth Israel Deaconess Medical Center, Harvard Medical School Boston Massachusetts USA; ^4^ Division of Cardiology UCLA David Geffen School of Medicine Los Angeles California USA; ^5^ Department of Surgery University of California Irvine (UCI) School of Medicine Irvine California USA; ^6^ Division of Geriatric Medicine Clinic Barmelweid Erlinsbach Switzerland; ^7^ Biomedical Imaging Pennington Biomedical Research Center Baton Rouge Louisiana USA

**Keywords:** body mass index, cognition, diabetes, prediabetes

## Abstract

**Objective:**

This study aimed to examine the association of BMI with cognitive performance in individuals with diabetes or prediabetes.

**Methods:**

Among Diabetes Prevention Program Outcomes Study (DPPOS) participants, BMI was categorized as normal (< 25 kg/m^2^), overweight (25 to < 30 kg/m^2^), or obesity (≥ 30 kg/m^2^). Cognitive tests included the Brief Spanish English Verbal Learning Test (B‐SEVLT) and the Digit Symbol Substitution test (DSST). The relationship between BMI at DPPOS Year 8 (Y8) visit and cognitive test scores at Y8, Y10, and Y15 visits was ascertained via linear mixed models accounting for repeated measures. Analogous models related BMI to Modified Mini‐Mental State exam (3MS) score at Y15.

**Results:**

A total of 2285 participants (mean ± SD age 51.1 ± 10.0 years; 67.7% female; 31% with overweight; and 60% with obesity at DPPOS Y8) completed cognitive assessments. Those with overweight or obesity at Y8 had a slower decline in B‐SEVLT immediate and delayed recall, compared with those with normal BMI; 3MS performance was higher among individuals with overweight or obesity compared to those with normal BMI at Y15.

**Conclusions:**

Among individuals with prediabetes or diabetes in DPPOS, overweight or obesity was associated with slower decline in verbal learning and memory compared with those with normal BMI.

**Study Importance**

- What is already known?
  ○ Individuals with diabetes and prediabetes have worse cognitive performance than those with normal glucose tolerance. Among individuals with diabetes or prediabetes, the association between BMI and cognitive performance is controversial.
- What does this study add?
  ○ In the Diabetes Prevention Project Outcomes Study (DPPOS), higher BMI was related to slower decline in cognitive performance, independent of glycemia and cardiovascular risk factors.
- How might these results change the direction of research or the focus of clinical practice?
  ○ The mechanisms underlying relationships between adiposity and cognitive decline in prediabetes and diabetes are multifaceted and complex and thus worthy of further study.

## 1 Introduction

Individuals with prediabetes or type 2 diabetes, representing two‐thirds of the adult United States population [1], are at higher risk of cognitive impairment [2]. Overweight and obesity increase the risk for type 2 diabetes [3]. All of these chronic diseases are associated with an increased risk of adverse cerebrovascular outcomes, including ischemic stroke [4], and thus are expected to be associated with worse cognition. Yet the independent association between overweight or obesity and cognition in persons with prediabetes or type 2 diabetes is not well understood. Clinical trials in this area have shown mixed results to date; for example, one intentional behavior change intervention targeting weight loss among middle‐aged and older adults with diabetes—which showed a variety of benefits for the core metabolic issues of diabetes along with many complications—had complex associations with cognitive function, with benefits and even hints of possible harms that differed between specific participant groups [5]. At least two additional clinical trials and one observational study among persons with type 2 diabetes have suggested that greater adiposity may be associated with better cognitive performance [6, 7, 8]. A better understanding of how excess adiposity (typically quantified by the body mass index, BMI) influences cognitive function in prediabetes or type 2 diabetes could help to inform strategies for the prevention of cognitive decline during long‐term treatment of individuals with prediabetes or type 2 diabetes.

Unfortunately, studies of adiposity and cognition in the general population have reported mixed results [9], providing few clues to guide corresponding studies in prediabetes or diabetes [10]. Studies concentrating on midlife (i.e., before age 65) are largely consistent in suggesting that higher BMI is related to worse cognitive function cross‐sectionally and longitudinally [11, 12, 13], along with increased risk of Alzheimer's disease (AD) later in life [14]. However, some studies in those 65 years and older suggest that higher BMI is related to *better* cognitive performance [12, 15, 16] and *lower* risk of dementia or mild cognitive impairment [17]. Weight change has also shown age‐dependent associations with cognition, with one observational study of older adults suggesting that weight loss is associated with poorer cognitive performance [18], and another study in middle‐aged adults finding no such association [13]. Clarifying the seemingly age‐dependent relationship between obesity and cognition remains important because obesity is modifiable, and a complete accounting of the risks and benefits of excess adiposity and weight loss throughout the life‐span is essential for guiding both antiobesity interventions and mechanistic studies.

While the association of adiposity and cognition has been assessed in the context of diabetes, to our knowledge no studies to date on the association between adiposity and cognition have focused on prediabetes, which affects almost 100 million adults in the United States [1]. Prediabetes represents a critical treatment window to prevent the development of diabetes, and it represents a transition state metabolically. It is currently not known whether excess adiposity in the context of this transition state incurs additional cognitive risks or protections for the individual; specifically, the cognitive risks and benefits associated with weight loss during prediabetes are not fully understood. The lack of studies of prediabetes with long‐term follow‐up (e.g., over the course of decades) is an especially critical gap, given the evidence of differences in the association depending on the relative time frame of adiposity exposures and cognitive outcomes.

In this hypothesis‐generating analysis, we examined associations between BMI categories of overweight and obesity, measured repeatedly in midlife and late life, with contemporaneous cognitive performance among participants in the Diabetes Prevention Program Outcomes Study (DPPOS) [19], a long‐term observational follow‐up of a clinical trial that enrolled adults with prediabetes and tracked them longitudinally for over two decades.

## 2 Methods

### 2.1 Cohort

The eligibility criteria, design, and methods of the Diabetes Prevention Program (DPP) [20] and DPPOS [21] have been reported elsewhere. Briefly, the DPP was a randomized trial comparing the effects of an intensive lifestyle intervention (ILS), metformin, and placebo on diabetes incidence among 3234 participants enrolled between 1996 and 1999 who were at high risk to develop diabetes. At entry, participants were required to have BMI ≥ 24 kg/m^2^ (≥ 22 kg/m^2^ in Asian Americans), fasting plasma glucose levels between 95 and 125 mg/dL, and impaired glucose tolerance (2‐h post‐load glucose of 140–199 mg/dL). People were excluded if taking medications known to alter glycemia or if they had illnesses that could reduce their life expectancy or their ability to participate in the trial. Written informed consent was obtained from all participants before screening, consistent with the Declaration of Helsinki and the guidelines of each center's institutional review board. Masked treatment was discontinued in July 2001, after an average intervention duration of 3.2 years. Following a 13‐month bridge period, during which all DPPOS participants were offered group‐implemented lifestyle intervention and the metformin arm was offered an open‐label extension of metformin treatment, all active DPP participants were invited to join DPPOS.

This is a repeated measures analysis of participants enrolled in DPPOS who completed cognitive assessments [19]. Analyses included 2285 DPPOS participants who provided BMI and complete cognitive assessment data at DPPOS Year 8, Year 10, and Year 15 visits. DPPOS Year 8 visits occurred between July 2009 and October 2010, DPPOS Year 10 visits occurred between July 2011 and October 2012, and DPPOS Year 15 visits occurred between July 2017 and October 2018; these visits occurred approximately 12, 14, and 19 years after DPP randomization.

### 2.2 Independent Variables: BMI Categories

BMI was calculated as weight in kilograms (kg) divided by height in meters squared (m^2^). Higher BMI indicates greater adiposity. Individuals were placed in BMI categories including normal (BMI < 25 kg/m^2^), overweight (25 to < 30 kg/m^2^), and obesity (BMI ≥ 30 kg/m^2^), following National Heart, Lung, and Blood Institute guidelines [22].

### 2.3 Dependent Variables: Cognitive Measures

The cognitive battery measured verbal learning and memory, along with frontal‐executive abilities. Learning and memory refer to the ability to acquire and recollect information [23], while frontal‐executive abilities refer to those necessary for planning and executing complex tasks and involve aspects such as psychomotor speed and attention [24]. All tests were administered in English or Spanish by centrally trained research staff according to the participant's reported first language. The measure of verbal learning and memory was the Spanish English Verbal Learning Test (B‐SEVLT) [25]. The B‐SEVLT consists of recalling a list of 15 words in three trials of immediate recall and one trial after a distractor list. For the B‐SEVLT, we examined two outcomes: the sum of the number of words recalled in the first three trials (immediate recall; B‐SEVLT‐IR) and the score of the fourth trial after the distractor list (delayed recall; B‐SEVLT‐DR). The test of frontal‐executive abilities was the total score in the Digit Symbol Substitution Test (DSST) [26]. The DSST is a test in which participants try to match numbers to symbols in 90 s. The total number of correct answers is reported. Global cognitive performance was assessed using the Modified Mini‐Mental State examination (3MS) [27]. The B‐SEVLT and DSST were administered in DPPOS Years 8, 10, and 15. The 3MS was administered once in Year 15. For all cognitive tests, a higher score indicates better performance.

### 2.4 Statistical Analysis

Baseline and follow‐up characteristics were compared between the three BMI categories using Pearson's chi‐square test or Fisher's exact test (when numbers were small) for categorical variables and ANOVA or Wilcoxon/Kruskal–Wallis test (when distribution was not normal) for continuous variables. Linear mixed models accounting for repeated measures from the same participants were conducted to assess changing rates over time from DPPOS Year 8 to Year 15 in B‐SEVLT‐IR, B‐SEVLT‐DR, and DSST between individuals whose BMI was categorized as normal, overweight, and obesity at Year 8, in three sequential models. Model 1 adjusted for treatment arms, BMI category at DPPOS Year 8, time measured in number of years in DPPOS (i.e., 8, 10 and 15 for the three waves of cognitive measurements), and interactions between time and BMI category. Model 2 additionally adjusted for age, sex, race/ethnicity, number of years of formal education level (< 12 vs. ≥ 12 years), and apolipoprotein E (APOE) ε4 genotype. Model 3 adjusts for systolic blood pressure (SBP), diastolic blood pressure (DBP), glycated hemoglobin (HbA1c), and low‐density lipoprotein cholesterol (LDL‐C) at DPPOS Year 8 in addition to the covariates in Model 2. Three analogous sequential linear models, without repeated measures, were estimated with the single 3MS measurement at DPPOS Year 15 as the outcome; these models included all terms described except those involving time from baseline.

Sensitivity analyses sought to explore the effects of possible confounders on these primary analyses. Four analyses sought to better understand the basis for BMI effects on cognition. One analysis used the BMI category concurrent with the cognitive outcome measurement years (i.e., DPPOS Years 8, 10, and 15) as the primary predictor of interest rather than the single measure of BMI category at Year 8. The second model replaced the BMI category at Years 8, 10, and 15 with a new BMI category calculated from the mean BMI across Years 8, 10, and 15. The third model added weight change from the DPP baseline to Year 8 as a covariate to assess the degree to which weight change associated with the DPP intervention could have accounted for the results. The fourth model categorized each individual at each time point as a BMI increaser or decreaser, that is, one whose BMI category became heavier (e.g., increased from overweight to obesity), became lighter (e.g., decreased from overweight to normal), or stayed the same between the previous measurement time point and the current one. This BMI change category replaced BMI as the primary predictor of cognitive change to assess the hypothesis that greater BMI decreases over time are associated with worse trajectories of cognitive change. Note that a linear trajectory analysis of individual BMI as a function of time was nonsignificant, suggesting there was inadequate evidence for systematic linear changes in BMI within individuals across measurement time points; this precluded the use of BMI linear trajectories in the sensitivity analyses. Although Model 2 adjusted for age, sensitivity analyses again assessed age as a confounder because older age is associated with poorer cognitive function generally, and because older age was associated with lower BMI in this cohort. Specifically, one additional analysis added a three‐way interaction term between time, BMI, and age stratum at the Year 8 assessment (age less than 55, 55–70, and over 70) to Model 2 as a covariate. Additional models included interaction terms between the BMI category and candidate effect modifiers including DPP treatment arm, sex, APOE carrier status, and race/ethnicity. Finally, an additional analysis adjusted for overall health‐related quality of life. The 36‐item Short‐Form Health Survey (SF‐36) is a widely used measure of health‐related quality of life [28, 29, 30, 31], and the Short Form 6D (SF‐6D) is a set of six items extracted from the SF‐36 [32]. The health‐related quality of life analysis included the SF‐6D summary score as an additional covariate. All analyses were completed in SAS 9.4.

## 3 Results

The analytic sample had a mean age of 63.2 years at Year 8 (Table 1). Two‐thirds of the sample were women, 54% were non‐Hispanic White, and 26.5% were homozygous or heterozygous for the APOE‐ε4 allele, the most widely replicated genetic risk factor for AD [33]. Participants with normal BMI were older than those with overweight and obesity. Participants with obesity were more likely to be female. The associations of many of the other variables in Table 1 with BMI group are thus confounded by age and sex.

**TABLE 1:** Comparisons of the characteristics of the DPPOS cohort that underwent cognitive testing at Years 8, 10, and 15 across BMI categories.

	All	Normal	Overweight	Obesity
Age at Randomization (years)	51.1 (9.95)	57.16 (10.04)	54.35 (10.17)	48.62 (8.92)
Age at DPPOS Y8 (years)	63.2 (9.86)	69.20 (9.81)	66.41 (10.14)	60.63 (8.80)
Year 8				
Sample size (N)	2285	210	696	1379
Female sex (number, percent)	1546 (67.7%)	119 (56.7%)	404/696 (58.0%)	1023/1379 (74.2%)
Ethnic and racial group (number, percent)				
White	1247 (54.6%)	127/210 (60.5%)	379/696 (54.5%)	741/1379 (53.7%)
African American	471 (20.6%)	24/210 (11.4%)	132/696 (19.0%)	315/1379 (22.8%)
Hispanic	334 (14.6%)	25/210 (11.9%)	98/696 (14.1%)	211/1379 (15.3%)
American Indian	128 (5.6%)	8/210 (3.8%)	37/696 (5.3%)	83/1379 (6.0%)
Asian	105 (4.6%)	26/210 (12.4%)	50/696 (7.2%)	29/1379 (2.1%)
Education > 12years (number, percent)	606 (26.5%)	78/210 (37.1%)	186/696 (26.7%)	342/1379 (24.8%)
APOE‐e4 (number, percent)	506 (26.5%)	38/168 (22.6%)	137/567 (24.2%)	331/1174 (28.2%)
HbA1c (percentage)	6.05 (1.03)	5.97 (0.88)	6.18 (1.01)	6.66 (1.28)
Systolic Blood pressure (mm Hg)	121 (14.1)	118.3 (13.72)	119.1 (14.53)	121.9 (13.45)
Diastolic Blood pressure (mm Hg)	71.4 (9.26)	67.61 (8.78)	69.96 (9.30)	73.24 (9.35)
Depression Score	4.52 (5.04)	4.57 (5.30)	3.86 (4.56)	4.84 (5.20)
Physical activity (MET‐hrs/wk)	16.1 (18.6)	21.15 (19.89)	19.61 (20.18)	13.52 (17.16)
Weight (kg)	91.2 (20.3)	65.51 (8.83)	78.23 (9.51)	101.6 (18.30)
Persons with diabetes (number, percent)	1104 (48.3%)	128/210 (61.0%)	370/696 (53.2%)	606/1379 (43.9%)
Duration of diabetes (years)	3.70 (4.33)	3.15 (4.52)	3.38 (4.27)	3.94 (4.32)
B‐SEVLT: sum of first 3 trials	26.5 (6.33)	25.14 (7.10)	25.53 (6.63)	27.26 (5.94)
B‐SEVLT_delay	9.89 (2.99)	9.07 (3.27)	9.47 (3.14)	10.23 (2.80)
DSST	49.2 (12.6)	46.43 (12.63)	46.98 (13.15)	50.82 (11.98)
Year 10				
Sample size (number)	2253	235	686	1332
Female sex (number, percent)	1536 (68.2%)	140/235 (59.6%)	401/686 (58.5%)	995/1332 (74.7%)
Ethnic and racial group (number, percent)				
White	1208 (53.6%)	139/235 (59.1%)	380/686 (55.4%)	689/1332 (51.7%)
African American	467 (20.7%)	31/235 (13.2%)	133/686 (19.4%)	303/1332 (22.7%)
Hispanic	336 (14.9%)	29/235 (12.3%)	90/686 (13.1%)	217/1332 (16.3%)
Asian	108 (4.8%)	28/235 (11.9%)	47/686 (6.9%)	33/1332 (2.5%)
American Indian	134 (5.9%)	8/235 (3.4%)	36/686 (5.2%)	90/1332 (6.8%)
Education > 12years (number, percent)	592 (26.3%)	72/235 (30.6%)	192/686 (28.0%)	328/1332 (24.6%)
APOE‐e4 (number, percent)	494 (26.1%)	54/190 (28.4%)	130/559 (23.3%)	310/1144 (27.1%)
HbA1c (percentage)	6.14 (1.17)	6.26 (1.42)	6.31 (1.19)	6.74 (1.35)
Systolic Blood pressure (mm Hg)	121 (14.9)	120.3 (14.65)	121.2 (16.05)	122.6 (15.00)
Diastolic Blood pressure (mm Hg)	70.9 (9.56)	67.19 (9.33)	70.15 (9.48)	72.50 (9.20)
Depression Score	4.63 (5.06)	4.88 (5.33)	4.11 (4.73)	4.86 (5.15)
Physical activity (MET‐hrs/wk)	15.0 (17.6)	21.14 (23.96)	16.90 (16.54)	12.88 (16.46)
Weight (kg)	90.4 (19.9)	65.14 (8.44)	77.97 (9.70)	100.8 (17.71)
Persons with diabetes (number, percent)	988 (43.9%)	127/235 (54.0%)	340/686 (49.6%)	521/1332 (39.1%)
Duration of diabetes (years)	4.78 (5.16)	4.25 (5.34)	4.33 (5.15)	5.11 (5.11)
B‐SEVLT: sum of first 3 trials	27.1 (6.54)	24.43 (7.20)	26.04 (6.89)	28.06 (6.01)
B‐SEVLT_delay	10.0 (3.02)	9.00 (3.36)	9.59 (3.13)	10.46 (2.82)
DSST	49.2 (13.0)	43.72 (13.38)	47.56 (12.72)	50.91 (12.64)
Year 15				
sample size (Number)	2112	350	605	1157
Female sex (number, percent)	1464 (69.3%)	224/350 (64.0%)	365/605 (60.3%)	875/1157 (75.6%)
Ethnic and racial group (number, percent)				
White	1100 (52.1%)	201/350 (57.4%)	311/605 (51.4%)	588/1157 (50.8%)
African American	442 (20.9%)	69/350 (19.7%)	110/605 (18.2%)	263/1157 (22.7%)
Hispanic	328 (15.5%)	39/350 (11.1%)	97/605 (16.0%)	192/1157 (16.6%)
Asian	101 (4.8%)	27/350 (7.7%)	44/605 (7.3%)	30/1157 (2.6%)
American Indian	141 (6.7%)	14/350 (4.0%)	43/605 (7.1%)	84/1157 (7.3%)
Education > 12years (number, percent)	558 (26.4%)	109/350 (31.1%)	169/605 (27.9%)	280/1157 (24.2%)
APOE‐e4 (number, percent)	450 (25.3%)	69/296 (23.3%)	123/484 (25.4%)	258/1000 (25.8%)
HbA1c (percentage)	6.43 (1.28)	6.04 (1.07)	6.26 (1.23)	6.59 (1.31)
Systolic Blood pressure (mm Hg)	123 (14.7)	120.8 (15.38)	123.0 (15.47)	123.9 (14.16)
Diastolic Blood pressure (mm Hg)	71.3 (9.73)	67.61 (9.09)	70.41 (9.74)	72.47 (9.62)
Weight (kg)	89.3 (19.9)	64.87 (8.83)	77.76 (9.63)	100.2 (17.53)
Persons with diabetes (number, percent)	766 (36.3%)	151/350 (43.1%)	243/605 (40.2%)	372/1157 (32.2%)
Duration of diabetes (years)	7.70 (7.18)	7.31 (7.47)	7.16 (7.20)	8.10 (7.06)
B‐SEVLT: sum of first 3 trials	25.2 (6.68)	22.21 (7.18)	24.64 (6.66)	26.00 (6.40)
B‐SEVLT_delay	9.28 (3.31)	7.89 (3.36)	9.03 (3.24)	9.68 (3.25)
DSST	46.1 (12.9)	41.02 (12.89)	45.18 (12.71)	47.51 (12.64)
3MSE	93.7 (8.01)	90.02 (13.48)	93.59 (7.64)	94.57 (6.34)

**FIGURE 1:**
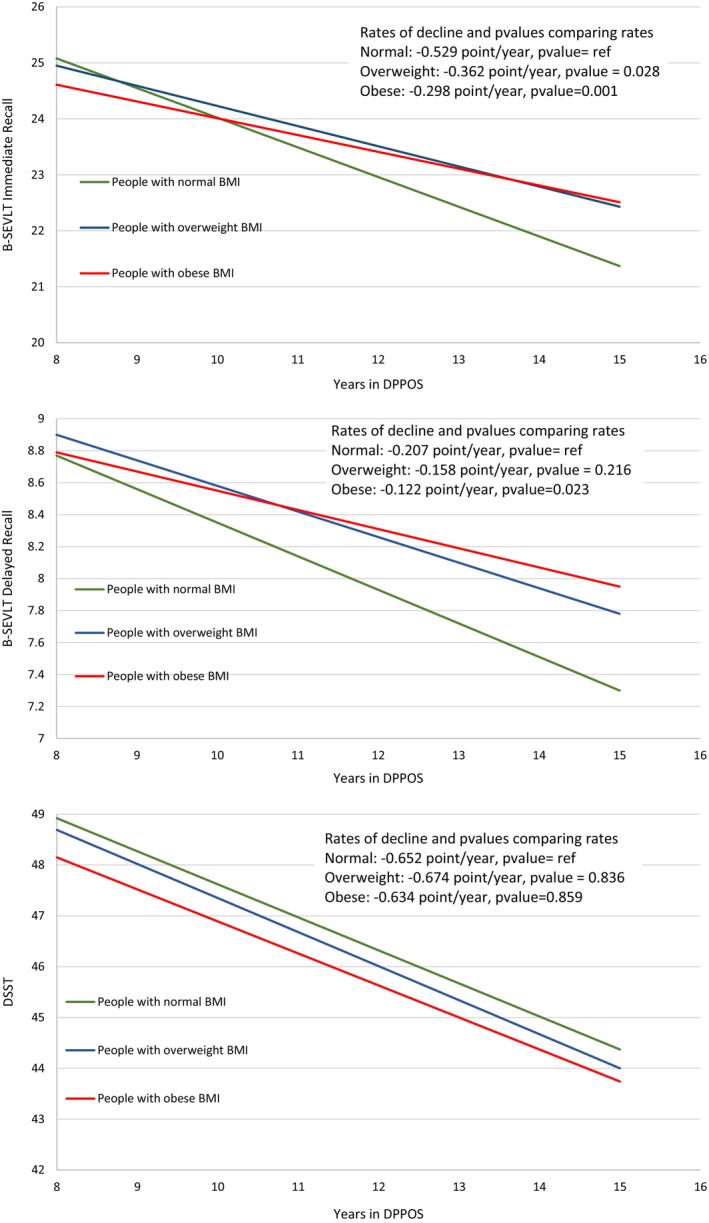
Estimated mean scores based on fitting straight lines through the results from DPPOS Years 8, 10, and 15. The results are derived in Tables 2 and 3. [Color figure can be viewed at wileyonlinelibrary.com]

Tables 2 and 3 shows the results of mixed models relating BMI categories to change in B‐SELVT‐IR, B‐SEVLT‐DR, and DSST. In models adjusting for demographics and randomization group, both B‐SEVLT performance measures declined significantly over time. Participants with overweight and obesity at Year 8 had less rapid decline in B‐SEVLT‐IR and B‐SEVLT‐DR than participants with normal BMI at Year 8 (Tables 2 and 3; Figure 1). For example, the mean changes in B‐SEVLT‐IR scores were −0.53, −0.36, and −0.30 per year in the normal, overweight, and obesity categories, respectively. These results were partially attenuated in a model adjusting for cardiometabolic covariates (Model 3; results not shown). There was no evidence of association between BMI category at Year 8 and change in DSST. Table 4 shows the results of linear regression models relating BMI categories at Year 15 to 3MS at Year 15. Performance was higher within the overweight and obesity groups compared to the group with normal BMI, and this result was robust across all models. While there was nominally better performance in the ILS and metformin groups than placebo among individuals with normal BMI, this difference was not statistically significant.

**TABLE 2:** Linear mixed model relating overweight and obesity status at DPPOS Year 8 to performance in immediate recall of the Spanish English Verbal Learning Test (B‐SEVLT), delayed recall of the B‐SEVLT, and total score in the Digit Symbol Substitution Test (DSST) at DPPOS Year 8/10/15.

	Coefficient	SE	*p*	95% CI
B‐SEVLT immediate recall				
Persons with overweight versus normal	−1.490	0.888	0.094	[−3.230, 0.251]
Persons with obesity versus normal	−2.309	0.844	0.006	[−3.962, −0.655]
Time (years)	−0.529	0.067	< 0.0001	[−0.661, −0.397]
Persons with overweight*time	0.167	0.076	0.028	[0.018, 0.316]
Persons with obesity*time	0.231	0.072	0.001	[0.091, 0.371]
B‐SEVLT delayed recall				
Persons with overweight versus normal	−0.269	0.444	0.544	[−1.139, 0.600]
Persons with obesity versus normal	−0.698	0.421	0.098	[−1.523, 0.128]
Time (years)	−0.207	0.035	< 0.0001	[−0.275, −0.138]
Persons with overweight*time	0.049	0.039	0.216	[−0.028, 0.126]
Persons with obesity*time	0.084	0.037	0.023	[0.012, 0.157]
DSST				
Persons with overweight versus normal	−0.073	1.416	0.959	[−2.849, 2.702]
Persons with obesity versus normal	−0.926	1.354	0.494	[−3.579, 1.727]
Time (years)	−0.652	0.094	< 0.0001	[−0.836, −0.468]
Persons with overweight*time	−0.022	0.106	0.836	[−0.230, 0.186]
Persons with obesity*time	0.018	0.100	0.859	[−0.178, 0.213]

*Note*: The model adjusts for treatment, time, age, sex, race, education level, and APOE genotype.

**TABLE 3:** Models in Table 2 evaluated for the groups with normal weight, overweight, and obesity at times 8, 10, and 15 (plotted in Figure 1).

	DPPOS year			Slope/year
	8	10	15	
B‐SEVLT immediate recall				
Normal	25.08	24.02	21.37	−0.529
Persons with overweight	24.95	24.23	22.43	−0.362
Persons with obesity	24.61	24.01	22.51	−0.298
B‐SEVLT delayed recall				
Normal	8.77	8.35	7.30	−0.207
Persons with overweight	8.90	8.58	7.78	−0.158
Persons with obesity	8.79	8.55	7.95	−0.122
DSST				
Normal	48.92	47.62	44.37	−0.652
Persons with overweight	48.69	47.35	44.00	−0.674
Persons with obesity	48.15	46.89	43.74	−0.634

**TABLE 4:** Relation of BMI categories with 3MS at DPPOS Year 15 from linear regression.

	Coefficient	SE	*p*	CI
Normal	—	—	—	
Persons with overweight	1.818	0.589	0.002	[0.663, 2.973]
Persons with obesity	1.655	0.570	0.004	[0.538, 2.773]

Lastly, we conducted two sets of sensitivity analyses: first, multiple imputation was employed to assess potential bias associated with missing data; second, marginal structural models were employed by using inverse probability of selection weights to reweight the sample to represent the original trial sample. The directions and sizes of the observed associations remain the same in the sensitivity analyses, although the standard errors are much larger due to the multiple imputation procedure. The associations estimated from the inverse‐selection‐probability weighted models remain in the same direction, with similar sizes (Table S3). In summary, the results are robust even in the presence of partially missing data.

None of the sensitivity analyses described in the *Methods* section substantively modified the general pattern of findings. In the model with only Year 8 BMI as the primary predictor of interest, those with overweight or obesity still had slower rates of B‐SEVLT‐IR and B‐SEVLT‐DR declines than those with normal BMI, and rates of DSST decline did not significantly differ by BMI category. Including the BMI category of the mean BMI across visits 8, 10, and 15 did not substantively change the results either. Weight change from the DPP baseline visit to the Year 8 visit was not a significant covariate in the models that included it, and none of the other estimated parameters was substantively changed by the inclusion of this covariate. Those whose BMI category dropped from obesity to overweight or from overweight to normal from the previous time point to the current one went on to have faster subsequent declines in B‐SEVLT immediate recall and B‐SEVLT delayed recall but not DSST (Table S2). The three‐way interaction term between BMI category, age stratum, and time was statistically significant in models for B‐SEVLT‐IR (*p* < 0.0001) and B‐SEVLT‐DR (*p* = 0.0019), as well as DSST (*p* < 0.0001); for B‐SEVLT‐IR and B‐SEVLT‐DR, the pattern of slower decline among individuals with overweight or obesity was especially prominent among those in the middle‐ and older‐age strata and attenuated among those in the youngest age stratum (Table 5). For DSST, the pattern of slower decline among individuals with overweight or obesity was only prominent within the oldest age stratum (Table 5). For 3MS, the differences between the overweight group and the normal group did not differ significantly across the age groups (*p* for age group*overweight = 0.2987); there were similar patterns for differences between the obesity and the normal groups (*p* for age group*obesity = 0.6437). The pattern of slower cognitive decline among individuals with overweight and obesity was not modified by adding any of the following as covariates to Model 2: a quadratic age term; the interaction of BMI category and DPP treatment arm; the interaction of BMI category and sex; the interaction of BMI category and APOE carrier status; the interaction of BMI category and race/ethnicity; and the SF6D summary score.

**TABLE 5:** Age group stratified analysis of performance in immediate recall of the Spanish English Verbal Learning Test (B‐SEVLT), delayed recall of the B‐SEVLT, and total score in the Digit Symbol Substitution Test (DSST) with respect to overweight and obesity status.

	Age at DPPOS Y8 < 55					55 ≤ Age at DPPOS Y8 < 70					Age at DPPOS Y8 ≥ 70				
	Coefficient	SE	*p*	95% CI		Coefficient	SE	*p*	95% CI		Coefficient	SE	*p*	95% CI	
B‐SEVLT immediate recall (*p* value for age_group*BMI status*time < 0.0001)															
Overweight versus normal	1.137	2.329	0.626	−3.428	5.701	−1.163	1.186	0.327	−3.487	1.161	−4.240	1.469	0.004	−7.119	−1.360
Obesity versus normal	2.074	2.155	0.336	−2.150	6.298	−1.839	1.099	0.095	−3.993	0.316	−3.357	1.517	0.027	−6.331	−0.383
Time (years)	−0.209	0.174	0.231	−0.550	0.132	−0.442	0.090	< 0.0001	−0.619	−0.265	−0.876	0.101	< 0.0001	−1.073	−0.678
Overweight*time	−0.007	0.195	0.972	−0.388	0.375	0.148	0.104	0.158	−0.057	0.352	0.393	0.126	0.002	0.146	0.640
Obesity*time	−0.047	0.178	0.792	−0.397	0.302	0.174	0.096	0.069	−0.013	0.362	0.333	0.127	0.009	0.084	0.583
B‐SEVLT delayed recall (*p* value for age_group*BMI status*time = 0.0019)															
Overweight versus normal	0.460	1.153	0.690	−1.800	2.719	−0.946	0.600	0.115	−2.122	0.229	−0.871	0.740	0.240	−2.321	0.579
Obesity versus normal	0.948	1.070	0.376	−1.149	3.044	−1.151	0.554	0.038	−2.237	−0.065	−0.751	0.752	0.319	−2.225	0.723
Time (years)	−0.040	0.087	0.651	−0.211	0.132	−0.230	0.046	< 0.0001	−0.320	−0.141	−0.330	0.053	< 0.0001	−0.434	−0.0227
Overweight*time	−0.033	0.098	0.738	−0.225	0.159	0.108	0.053	0.043	0.004	0.211	0.100	0.066	0.131	−0.030	0.230
Obesity*time	−0.049	0.090	0.586	−0.225	0.127	0.121	0.048	0.012	0.026	0.216	0.096	0.067	0.152	−0.035	0.226
DSST (*p* value for age_group*BMI status*time < 0.0001)															
Overweight versus normal	2.864	3.823	0.454	−4.630	10.358	−0.128	1.704	0.940	−3.468	3.213	−2.668	1.830	0.146	−6.254	0.919
Obesity versus normal	4.650	3.554	0.191	−2.315	11.615	−0.344	1.612	0.831	−3.504	2.816	−1.929	1.887	0.307	−5.628	1.770
Time (years)	−0.429	0.285	0.132	−0.987	0.129	−0.606	0.127	< 0.0001	−0.856	−0.356	−1.076	0.123	< 0.0001	−1.317	−0.834
Overweight*time	−0.027	0.319	0.934	−0.652	0.599	0.006	0.147	0.968	−0.283	0.295	0.262	0.157	0.096	−0.046	0.569
Obesity*time	−0.107	0.292	0.715	−0.679	0.466	−0.065	0.135	0.630	−0.329	0.199	0.194	0.156	0.213	−0.111	0.499

*Note*: The interactions between overweight and time represent differences in the rates of change over time in the B‐SEVLT immediate recall, B‐SEVLT delayed recall, and DSST between the overweight and normal groups. We can see that the differences are all negative in the age at Year 8 less than 55 subcohort, while the differences are almost all positive in the other two age groups. Therefore, the rates of decline in cognitive scores in the overweight and obesity groups are faster when participants are less than 55 at Year 8, but the rates of decline among persons with overweight and persons with obesity were slower when participants were over 55 at Year 8. The differences between the opposite patterns between different age groups are statistically significant.

## 4 Discussion

In this longitudinal analysis among participants with prediabetes or diabetes in the DPPOS, we found that overweight or obesity was associated with a slower decline in verbal learning and memory. There was also a similar cross‐sectional association between overweight or obesity and better performance on global cognition assessed with the 3MS. These findings were especially pronounced among the oldest participants in the study. Together, these results add to a mixed literature in which associations between adiposity and cognitive function appear to vary depending on when in the life‐span the two constructs are measured, the physical health and cognitive status of the individual, and other factors. They also underscore the need for a greater knowledge of the mechanisms through which adiposity influences cognitive functioning in midlife and old age. The overall implications of these findings are that, despite widespread agreement that elevated BMI confers a higher risk of adverse cardiometabolic outcomes in prediabetes and diabetes, the relationship between BMI and cognitive functioning in this setting is complex.

It is possible that our findings are a by‐product of our use of BMI, whose limitations as an indirect measure of adiposity are well documented. Yet our findings are consistent with those of prior studies that used alternative measures of adiposity. For example, an analysis in the Cardiovascular Health Study in individuals with and without diabetes aged 65 years and older found that BMI, waist circumference, waist‐to‐hip ratio, and bioelectrical‐impedance‐based fat mass were related to better cognitive performance cross‐sectionally and longitudinally [15], consistent with our finding in a younger sample with prediabetes and diabetes. A cross‐sectional analysis of the Glycemia Reduction Approaches in Diabetes: A Comparative Effectiveness Study (GRADE) in a cohort of 5018 individuals with type 2 diabetes with a mean age of 56.7 years reported that high BMI was also related to better performance in the B‐SEVLT that was independent of insulin sensitivity [7]. Future work should try to overcome limitations of these body composition assessment techniques by using more accurate and precise methods, such as dual‐emission X‐ray absorptiometry (DXA) or stable isotope tracers, to assess adiposity and its association with cognition among individuals with prediabetes and type 2 diabetes.

We must also consider potential factors that threaten the validity of our findings, including confounding, bias, and chance. Our results were robust across models adjusting for demographic, diabetes‐related, and cardiovascular confounders. Thus, it seems unlikely that our results were due to confounding, although residual or unmeasured confounding is possible. Our cohort was relatively young, and we expect that only a very small number of participants would have met clinical criteria for dementia; therefore, we believe it is unlikely that our results could be due to unintentional weight loss that is driven directly by clinical dementia [34]. While it is possible that our results are due to chance, particularly in a relatively large sample such as the one used for this report, the results remained robust in various analyses and models. Finally, given a lack of differences in BMI between individuals who *were* versus who *were not* censored between cognitive evaluations (Table S1), we believe our findings are unlikely to be explained by competing risk of censorship due to obesity‐related adverse outcomes. That said, we cannot fully discount the possibility that an unmeasured bias or confounder could have influenced results.

With all of these threats to validity in mind, we note that there have also been plausible proposals for mechanisms that could explain the association between higher BMI and better cognitive performance. The adipokine leptin, which increases with higher BMI, has been hypothesized to be neuroprotective [35]. The Framingham Heart Study reported that higher leptin levels were associated with a lower risk of cognitive impairment and higher brain volume, a surrogate marker of brain health [36]. Uric acid, the serum levels of which are positively correlated with BMI, has also been hypothesized to be neuroprotective [37]. Future work could explore the role these mechanisms could have played in our observed findings.

Another plausible mechanism is supported by our analysis that suggested that those whose BMI declined from the previous time point to the current one went on to have greater cognitive decline from the current time point to the subsequent one. Older adults can experience malnutrition driven by reduced access to healthy foods, as well as due to sensory and metabolic changes caused by multiple chronic diseases and the side effects of their treatments [38]. The same malnutrition, chronic diseases, and treatment side effects can lead to concomitant cognitive decline, which can in turn exacerbate attempts to consume a nutritious diet and manage chronic diseases. This interaction between chronic diseases and cognitive decline has led to reports of associations between unintentional late‐life weight loss (and clinical phenotypes such as frailty that feature unintentional weight loss as a component) and cognitive decline [39]. Our observation of an association between recent weight loss and subsequent cognitive decline is consistent with this association, although the inclusion of several variables representing chronic diseases in our models (including a summary indicator of comorbidity burden, the SF6D) did not substantially modify the pattern of findings. Future work should endeavor to disentangle the interconnected effects of malnutrition, chronic diseases, and their treatment on both adiposity and cognitive decline in samples like this one.

There are multiple strengths in this analysis. The cohort is large, well phenotyped, and diverse across a number of dimensions. The cohort has been followed longitudinally for over two decades, thus allowing characterization of cognitive change at multiple time points per individual over approximately 7 years. The tests of cognition are widely used and standardized.

The main limitation is that as a clinical trial cohort, DPPOS is unlikely to be representative of the general population. As a cohort of individuals who all had prediabetes and overweight or obesity at baseline, the cohort was relatively metabolically homogeneous at baseline; therefore, it is unclear how results would generalize to broader cohorts that include individuals who were metabolically healthy or diagnosed with diabetes at study entry.

## 5 Conclusion

Higher BMI at Year 8 was associated with slower cognitive decline in verbal learning and memory when reassessed approximately 7 years later among individuals with prediabetes or type 2 diabetes in the DPPOS, as reported in other studies. Additional research is needed to discover the mechanisms explaining the association between higher adiposity and better cognition in older age, particularly in the risk group with prediabetes and type 2 diabetes.

## Author Contributions

J.A.L. conceived of the research question. J.A.L. and Q.P. designed the analytic approach. Q.P. conducted the analysis, and J.A.L. and O.T.C. drafted the full manuscript for review by co‐authors. J.A.L., Q.P., W.C.K., M.M., K.W., K.M.G., M.S., and O.T.C. made substantial contributions to the interpretation of results and scientific review and revision of the full manuscript draft and provided final approval of the version to be published. Q.P. completed the analyses, advised on the interpretation of results, made significant contributions to scientific review and revision of the full manuscript draft, and provided final approval of the version to be published. O.T.C. is the guarantor of this work and, as such, had full access to all the data in the study and takes responsibility for the integrity of the data and the accuracy of the data analysis.

## Conflicts of Interest

J.A.L. was a consultant to Merck KGaA in 2022 and to Novo Nordisk in 2024 and receives a stipend from Wolters Kluwer as Editor in Chief of Alzheimer's Disease and Associated Disorders and royalties from Springer as co‐editor of the book Diabetes and the Brain. The other authors declared no conflicts of interest.

## Supporting information


**Table S1:** Sensitivity analysis of Tables 2 and 3. Model 1 adds HbA1c, SBP, DBP, and LDL to the main model in Tables 2 and 3 (demographics, education level, APOE, treatment, BMI status, time). Model 2 adds SF6D to model 1. Model 3 adds weight change from DPP baseline to DPPOS Y8/10/15 to model 2.
**Table S2:** Linear mixed models regressing performance in immediate of the Spanish English Verbal Learning Test (B‐SEVLT), delayed recall of the B‐SEVLT, and total score in Digit Symbol Substitution Test (DSST) at DPPOS Y8/10/15 using different forms of BMI status: change of BMI status from baseline to DPPOS Y8, DPPOS Y8 to DPPOS Y10, DPPOS Y10 to DPPOS Y15; concurrent BMI status at DPPOS Y8/10/15; BMI status based on the average weight of DPPOS Y8/10/15. The models adjust for treatment, time, age, sex, race, education level and APOE genotype.
**Table S3:** Comparison of sensitivity analysis (marginal structural models using inverse‐selection‐probability* weights) with the main analysis (complete case). *Probability of selection into the DPPOS Y8 subsample was estimated based on age, sex, race and ethnicity, number of years of education, DPP randomization arm, diabetes status and duration (if diabetic) at the end of follow‐up, and the following measurements taken at the time of DPP enrollment: fasting glucose, hemoglobin A1C, systolic and diastolic blood pressure, depression score, and average number of METs of leisure activity per week, household income, weight, BMI. Inverse probability weights were applied in the same regression models featured in our primary analysis.
**Figure S1:** Histogram of 3 MSE score by BMI category.

**Data S1:** oby70031‐sup‐0002‐InvestigatorsAppendix.docx.

## Data Availability

The data that support the findings of this study are openly available in NIDDK Data Repository at https://doi.org/10.58020/66x5‐8y21.
