# Supplementary material for: Association of BMI and Cognitive Performance in the Diabetes Prevention Program Outcomes Study
Source: Obesity (Silver Spring). 2025 Sep 28;33(12):2365–76. doi: 10.1002/oby.70031 (PMC12636062; doi:10.1002/oby.70031)
Supplement: Supplementary file 1 — Table S1: Sensitivity analysis of Tables 2 and 3. Model 1 adds HbA1c, SBP, DBP, and LDL to the main model in Tables 2 and 3 (demographics, education level, APOE, treatment, BMI status, time). Model 2 adds SF6D to model 1. Model 3 adds weight change from DPP baseline to DPPOS Y8/10/15 to model 2. Table S2: Linear mixed models regressing performance in immediate of the Spanish English Verbal Learning Test (B‐SEVLT), delayed recall of the B‐SEVLT, and total score in Digit Symbol Substitution Test (DSST) at DPPOS Y8/10/15 using different forms of BMI status: change of BMI status from baseline to DPPOS Y8, DPPOS Y8 to DPPOS Y10, DPPOS Y10 to DPPOS Y15; concurrent BMI status at DPPOS Y8/10/15; BMI status based on the average weight of DPPOS Y8/10/15. The models adjust for treatment, time, age, sex, race, education level and APOE genotype. Table S3: Comparison of sensitivity analysis (marginal structural models using inverse‐selection‐probability* weights) with the main analysis (complete case). *Probability of selection into the DPPOS Y8 subsample was estimated based on age, sex, race and ethnicity, number of years of education, DPP randomization arm, diabetes status and duration (if diabetic) at the end of follow‐up, and the following measurements taken at the time of DPP enrollment: fasting glucose, hemoglobin A1C, systolic and diastolic blood pressure, depression score, and average number of METs of leisure activity per week, household income, weight, BMI. Inverse probability weights were applied in the same regression models featured in our primary analysis. Figure S1: Histogram of 3 MSE score by BMI category. [file OBY-33-2365-s001.docx]

**Supplemental Table 1.** Sensitivity Analysis of Table 2. Model 1 adds HbA1c, SBP, DBP, LDL to the main model in Table 2 (demographics, education level, APOE, treatment, BMI status, time). Model 2 adds SF6D to model 1. Model 3 adds weight change from DPP baseline to OSY8/10/15 to model 2.

**Supplemental Table 2.** Linear mixed models regressing performance in immediate of the Spanish English Verbal Learning Test (B-SEVLT), delayed recall of the B-SEVLT, and total score in Digit Symbol Substitution Test (DSST) at OSY8/10/15 using different forms of BMI status: change of BMI status from baseline to OSY8, OSY8 to OSY10, OSY10 to OSY15; concurrent BMI status at OSY8/10/15; BMI status based on the average weight of OSY8/10/15. The models adjust for treatment, time, age, sex, race, education level and APOE genotype.

**Supplemental Table 3.** Comparison of sensitivity analysis (marginal structural models using inverse-selection-probability* weights) with the main analysis (complete case).

|  | **Coefficient (Original)** | **Coefficient (IPW)** | **SE (Original)** | **SE (IPW)** | **P value (Original)** | **P value (IPW)** | **95% CI (Original)** | | **95% CI (IPW)** | |
| --- | --- | --- | --- | --- | --- | --- | --- | --- | --- | --- |
| B-SEVLT immediate recall | | | | | | | | | | |
| Overweight vs. normal | -1.490 | -1.606 | 0.888 | 0.905 | 0.094 | 0.076 | -3.230 | 0.251 | -3.379 | 0.167 |
| Obese vs. normal | -2.309 | -2.579 | 0.844 | 0.857 | 0.006 | 0.003 | -3.962 | -0.655 | -4.259 | -0.899 |
| time(year) | -0.529 | -0.535 | 0.067 | 0.068 | <.0001 | <.0001 | -0.661 | -0.397 | -0.669 | -0.401 |
| Overweight* time | 0.167 | 0.168 | 0.076 | 0.077 | 0.028 | 0.030 | 0.018 | 0.316 | 0.016 | 0.320 |
| Obese*time | 0.231 | 0.242 | 0.072 | 0.073 | 0.001 | 0.001 | 0.091 | 0.371 | 0.100 | 0.384 |
| B-SEVLT delayed recall | | | | | | | | | | |
| Overweight vs. normal | -0.269 | -0.342 | 0.444 | 0.450 | 0.544 | 0.447 | -1.139 | 0.600 | -1.224 | 0.540 |
| Obese vs. normal | -0.698 | -0.765 | 0.421 | 0.426 | 0.098 | 0.073 | -1.523 | 0.128 | -1.599 | 0.070 |
| time(year) | -0.207 | -0.208 | 0.035 | 0.035 | <.0001 | <.0001 | -0.275 | -0.138 | -0.278 | -0.139 |
| Overweight* time | 0.049 | 0.051 | 0.039 | 0.040 | 0.216 | 0.206 | -0.028 | 0.126 | -0.028 | 0.129 |
| Obese*time | 0.084 | 0.084 | 0.037 | 0.038 | 0.023 | 0.025 | 0.012 | 0.157 | 0.010 | 0.157 |
| DSST | | | | | | | | | | |
| Overweight vs. normal | -0.073 | -0.250 | 1.416 | 1.453 | 0.959 | 0.864 | -2.849 | 2.702 | -3.097 | 2.598 |
| Obese vs. normal | -0.926 | -0.782 | 1.354 | 1.384 | 0.494 | 0.572 | -3.579 | 1.727 | -3.496 | 1.931 |
| time(year) | -0.652 | -0.639 | 0.094 | 0.096 | <.0001 | <.0001 | -0.836 | -0.468 | -0.828 | -0.451 |
| Overweight* time | -0.022 | -0.016 | 0.106 | 0.109 | 0.836 | 0.884 | -0.230 | 0.186 | -0.230 | 0.198 |
| Obese*time | 0.018 | 0.007 | 0.100 | 0.102 | 0.859 | 0.944 | -0.178 | 0.213 | -0.193 | 0.207 |

*Probability of selection into the DPPOS year 8 subsample was estimated based on age, sex, race and ethnicity, number of years of education, DPP randomization arm, diabetes status and duration (if diabetic) at the end of follow-up, and the following measurements taken at the time of DPP enrollment: fasting glucose, hemoglobin A1C, systolic and diastolic blood pressure, depression score, and average number of METs of leisure activity per week, household income, weight, BMI. Inverse probability weights were applied in the same regression models featured in our primary analysis

Supplemental Figure 1: Histogram of 3 MSE score by BMI category
